# Deep learning for complex chemical systems

**DOI:** 10.1093/nsr/nwad335

**Published:** 2023-12-29

**Authors:** Wei Li, Guoqiang Wang, Jing Ma

**Affiliations:** Key Laboratory of Mesoscopic Chemistry of Ministry of Education, Institute of Theoretical and Computational Chemistry, School of Chemistry and Chemical Engineering, Nanjing University, China; Key Laboratory of Mesoscopic Chemistry of Ministry of Education, Institute of Theoretical and Computational Chemistry, School of Chemistry and Chemical Engineering, Nanjing University, China; Key Laboratory of Mesoscopic Chemistry of Ministry of Education, Institute of Theoretical and Computational Chemistry, School of Chemistry and Chemical Engineering, Nanjing University, China

## Abstract

Deep learning forms a bridge between the local features of molecular fragments/localized orbitals and the global properties of complex systems, enabling multi-scale simulations of complex chemical systems and reaction processes.

Modern experimental techniques and theoretical simulations extended the research scope of complex chemical processes in functional molecules/materials innovation. Quantum chemistry (QC)-level simulations of macromolecules and condensed-phase systems need machine learning (ML) or deep learning (DL) [1] to decrease computational costs, which traditionally increases exponentially with system size. To guide rational design and optimize synthetic processes, we need to explore the sophisticated chemical reaction networks (CRN*s*), consisting of coupling pathways among reactants, catalysts, intermediates, solvents, and products.

Physical-informed and brain-inspired ML are increasingly employed to discover patterns hidden behind extensive computational and experimental data, explore the structure-property relationship, or accelerate calculations of energy, force, etc (Fig. 1). Unlike classical ML methods (Supplementary Table S1) using hand-crafted features, DL models (convolutional neural network, recurrent neural network, message passing neural network, etc) automatically learn underlying representations, increasing their popularity within the chemistry community (Supplementary Fig. S1). The DL models utilize nonlinear activation functions in the hidden layers to capture the nonlinear relationship between the input descriptors (e.g. molecular graph or fingerprints) and outputs (e.g. energy, forces, molecular properties) (Supplementary Table S2).

**Figure 1.**
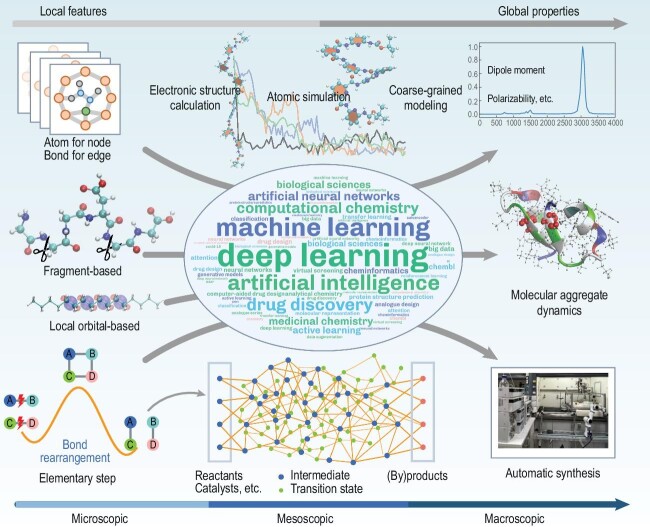
Deep learning in complex chemical systems.

DL models learn from the human brain to build interrelationships between memory and learning with adaptive responses to variable environments on various time scales. Building an intelligent neural network is as complex as solving intercoupling equations in theoretical chemistry. Using localized orbitals or fragments, local correlation or fragmentation approaches (Supplementary Table S3) [2] could treat the macromolecules consisting of hundreds of atoms at even electron correlation levels. Connecting electronic structure calculations to atomic simulations using a graph-based input of local atoms and bonds is similar to using fragments or localized orbitals in fragmentation [2,3] or local correlation [4] approaches for complex systems. Unsupervised learning, like dimensionality reduction, clustering, or density estimation, can extract crucial information from MD-simulated data. The successful prediction of global extensive (e.g. electronic energy, Gibbs free energy) or intensive (e.g. orbital energy, polarizability) properties is achieved by using graph convolutional models with atoms and bonds [5], like the atomic types in classical force fields (FFs). The awareness of the local chemical environment could be learned by message passing and attention mechanism (adaptive learning) [5], like self-consistent or optimization procedures.

ML-FFs [1] bridge the QC and atomic scales, enabling QC-level MD simulations at timescales comparable to classical FFs. Spatial-invariant descriptors are constructed to train nonlinear models for energy/forces prediction, employing kernel-based or DL models (Supplementary Table S4) and commonly used descriptors, including atomic-centered symmetry function, smooth overlap of atomic position, etc (Supplementary Table S5). The universal ML-FF, trained on small molecules by neglecting long-range interactions, often lacks high accuracy and requires a vast training set. The specific ML-FF for particular systems can theoretically attain higher accuracy but struggles with macromolecules and condensed phase systems due to large size and huge conformational space. An extension to the coarse-grained or mean-field treatment would realize the mesoscopic simulations of self-assembly or phase properties of soft matter [6]. Owing to the challenge in generalization of couplings between different electronic states, only some specific ML-FFs for excited states are available for small-sized systems and very few electronic states (Supplementary Table S6) [7].

When reaction modeling allows bond-breaking/formation, changes in atomic types, local bonding rearrangements, and electronic structure should be accurately learned within a CRN, unveiling reaction mechanisms and discovering new reactions at both microscopic and macroscopic levels. Exploring high-energy reaction regions on potential energy surfaces surpasses classical FF capabilities. This challenge has been alleviated by automated, unbiased reaction path-searching methods like metadynamics, combined MD and coordinate driving, and deep reinforcement learning (Supplementary Tables S7 and S8). For complex systems with numerous atoms or multiple reaction components, computational demands increase due to the combinatorial explosion. ML atomic potentials or reactive FFs could balance accuracy and efficiency for long-term, large-scale simulations and rare transition sampling. DL potentials like DeepMD [8] were applied to explore CRN involving heterogeneous catalysis and reactions in solution.

Combining simulations and ML with automatic chemical synthesis allows the creation of intelligent laboratories [9] for rapid discovery of reactions and functional materials (Supplementary Table S9). The target-oriented virtual screening aligns with the make-on-demand principle, reducing the time and costs of experimental trials. While some DL-assisted virtual screening has been employed in drug and materials discovery (Supplementary Table S10), the screening space could become overwhelmingly vast. Deep generative models and generative adversarial networks provide potential solutions.

The goal of DL is to start from microscopic simulation, span multiscales, and achieve macroscopic prediction. At the atomic level, fragment-based DL-FFs can predict large systems’ ground-state energy and forces through subsystems training [10], with potential extensions to excited states using density-like or auto-learning descriptors [7]. Adaptive coarse-grained ML-FF built by learning fragment properties is crucial for accurately capturing long-range interactions in mesoscopic simulations. At the macroscopic level, DL is essential for predicting properties and modeling reactions. The DL-FFs enable efficient simulation of complex reaction processes, combining with an automated reaction path search for data sampling in the transition state region. A knowledge-controlled CRN construction strategy, through DL-based reaction rules, can automatically filter reaction combinations and potential active sites, addressing the combinatorial explosion issue. DL-assisted reaction process modeling enables mapping of complete CRN in real chemical environments, and is being anticipated to be the next frontier. ML and automated experimentation can accelerate optimizing reaction parameters and synthesizing target molecules; however, discovering novel reactions remains rare. The multimodal and pre-trained DL models allow autonomous exploration of uncharted reactions and integration with automation technology to create a universal discovery platform. This links microscopic electronic configuration changes, mesoscopic molecular aggregation, and macroscopic property predictions and feedback. This platform requires expertise in DL algorithms and coding, thus urgently needing a user-friendly platform for easy parameter adjustments. The automatic experimentation can generate extensive standardized data, facilitating the development of forward/reverse reaction designs and target identification.

Understanding why, how, and when DL methods work is also instructive for solving complex chemical problems by sharing code and creating high-quality datasets. In turn, reducing the scaling of QC calculations aids in building an efficient DL model. Understanding the subtle interplay between the local and global properties is key to chemical theory and simulation models, highlighting the potential application of a sustainable synergistic platform of chemistry and DL to material and drug designs.

## Supplementary Material

nwad335_Supplemental_File
