# Supplementary material for: Deep learning for complex chemical systems
Source: Natl Sci Rev. 2023 Dec 29;10(12):nwad335. doi: 10.1093/nsr/nwad335 (PMC10808951; doi:10.1093/nsr/nwad335)
Supplement: nwad335_Supplemental_File [file nwad335_supplemental_file.pdf]

Supplementary Material

Deep Learning for Complex Chemical Systems

Wei Li, Guoqiang Wang, and Jing Ma*

Key Laboratory of Mesoscopic Chemistry of Ministry of Education, Institute of Theoretical and Computational Chemistry, School of Chemistry and Chemical Engineering, Nanjing University, Nanjing 210023, China

* Email: majing@nju.edu.cn

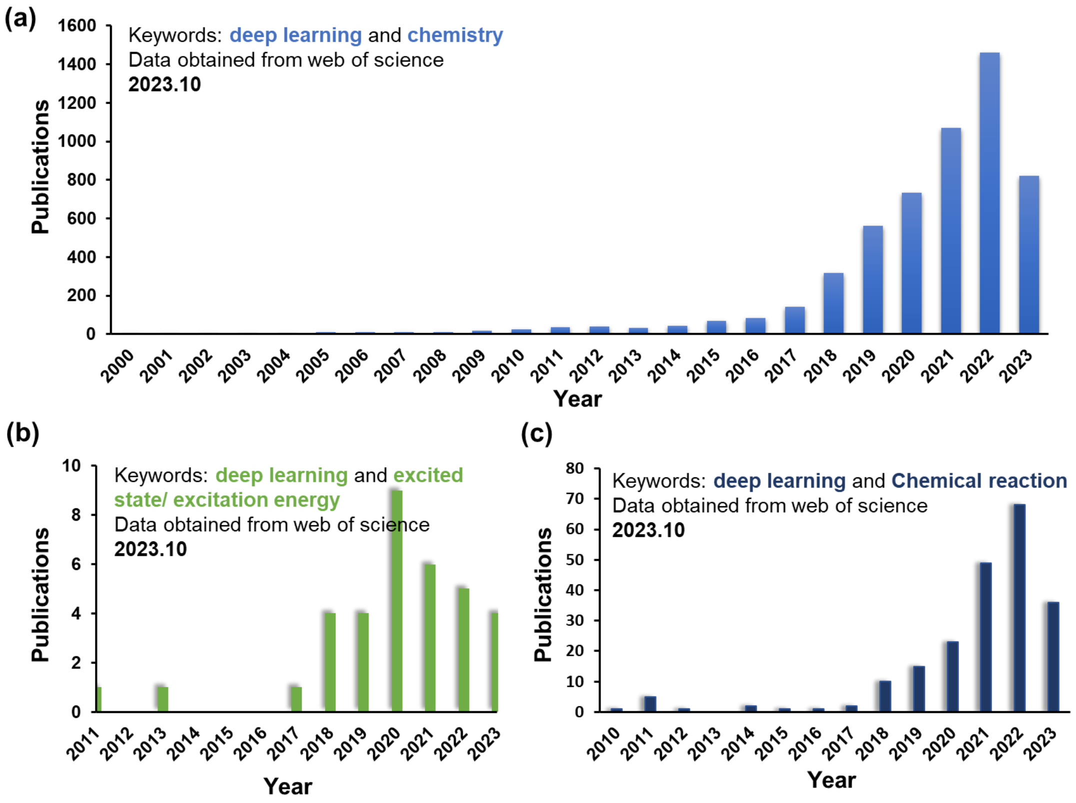

Figure S1. Annual publications in deep learning-related chemistry: (a) deep learning and chemistry; (b) deep learning and excited state/excitation energy; (c) deep learning and chemical reaction.

Table S1. Some classical machine learning methods

Method	Description
**Random Forest**	A machine learning algorithm using multiple random decision trees to predict a single result
**LightGBM**	A gradient-boosting framework that uses tree-based learning algorithms
**XGBoost**	Extreme Gradient Boosting (a distributed gradient-boosting decision tree)
**GBDT**	Gradient-boosted decision tree
**SVM**	Support vector machines
**……**	……

Table S2. Some deep learning methods to reduce computational costs of electronic structure (ES), such as density functional theory (DFT), second-order Møller-Plesset perturbation theory (MP2), coupled cluster singles and doubles (CCSD), CCSD with perturbative triples correction [CCSD(T)], etc.

Method	ES method	Target	Ref.
**HDNNP**	DFT	energy, forces	[1,2]
**Deep Potential**	DFT	energy, forces	[3,4]
**SchNet**	DFT	energy, forces, molecular properties	[5,6]
**PhysNet**	DFT	energy, forces, dipole moments, partial charges	[7]
**AimNet**	DFT	energy, atomic charges, solvation free energies	[8]
**MBE-NN**	RI-MP2	energy, forces	[9]
**EE2B-NN**	CCSD	energy, forces	[10]
**rSMF-NN**	DFT	energy, forces	[11]
**ML-MLEBF**	CASSCF	excited-state energy and forces	[12]
**NN-GMFCC**	DFT	energy, forces	[13,14]
**GEBF-NN**	DFT	energy, forces	[15–17]
**grid-based ML**	MP2	energy	[18]
**SchNOrb**	MP2	energy, atomic charges, dipole moments	[19]
**……**	……	……	……

Table S3. Linear scaling methods with the utilization of the locality of molecular fragments or localized orbitals

Method	ES method	Target	Ref.
**Fragment-based approach**			
**DC**	DFT, MP2, CCSD(T)	energy, forces (MP2)	[20–22]
**ELG**	MP2	energy	[23]
**GEBF**	DFT, MP2, CCSD(T), etc.	energy, forces, Hessians, etc.	[24,25]
**SMF**	DFT, MP2, CCSD(T), etc.	energy, forces, Hessians, etc.	[26]
**MTA**	DFT, MP2, CCSD(T), etc.	energy, forces, Hessians, etc.	[27]
**GMFCC**	DFT, MP2, CCSD(T), etc.	energy, forces, Hessians, etc.	[28,29]
**MIM**	DFT, MP2, CCSD(T), etc.	energy, forces, Hessians, etc.	[30]
**GMBE**	DFT, MP2, CCSD(T), etc.	energy, forces, Hessians, etc.	[31]
**……**	……	……	……
**Localized orbital-based approach**			
**LMP2, LCC**	MP2, CCSD, CCSD(T), etc.	energy, forces (MP2)	[32,33]
**CIM**	MP2, CCSD, CCSD(T), etc.	energy, forces (MP2)	[34–37]
**DLPNO**	MP2, CCSD, CCSD(T), etc.	energy, forces (MP2)	[38,39]
**PNO**	MP2, CCSD, CCSD(T), etc.	energy	[40]
**OSV**	MP2, CCSD, CCSD(T), etc.	energy, forces (MP2)	[41]
**Incremental**	MP2, CCSD, CCSD(T), etc.	energy	[42,43]
**LNO**	MP2, CCSD, CCSD(T), etc.	energy	[44,45]
**DEC**	MP2, CCSD, CCSD(T), etc.	energy, forces (MP2)	[46]
**……**	……	……	……

Table S4. Two typical kernel-based or deep learning-based machine learning force fields and their applications

Methods	1) Gaussian process regression (GPR)[47]<br>2) Multi-layer neural network[48]
Applications	3) Predict the relationships between molecular or material structures and properties<br>✓ Optical properties<br>✓ Electrical properties<br>✓ Magnetic properties<br>4) Construct accurate and efficient molecular potential energy surfaces (PESs) or intermolecular interaction potentials<br>✓ gas-phase dynamics simulations<br>✓ Molecular dynamics (MD) simulations<br>✓ Searching for chemical reaction pathways.

Table S5. Commonly used descriptors in machine learning force fields[49]

Descriptors	Description
ACSFs	atomic-centered symmetry functions
SOAP	smooth overlap of atomic positions

Table S6. Excited-state machine learning force field

Excited-state electronic structure methods	1) Time-dependent density functional theory (TDDFT)[50,51]<br>2) Wavefunction theory (WFT)[52–54]
Properties predicted in machine learning[55]	3) Electronic energies<br>4) Gradients<br>5) Spin-orbit couplings<br>6) Nonadiabatic couplings<br>7) Permanent and transition dipole moment
Challenges and possible solutions	8) Expensive for large systems, requiring linear scaling excited-state algorithms<br>9) Phase factor problem in electronic wavefunction (transition dipole moments are obtained from two electronic states), requiring phase correction[56]

Table S7. Selected examples of computational tools for the exploration of complex reaction networks.[a]

**Computational method**	**Ref.**
Metadynamics	57
Combined molecular dynamics and coordinate (MD/CD)	58
Deep reinforcement learning	59
......	......
[a]For the reaction pathway searching strategies, one can see a comprehensive review by Zimmerman and co-workers.[60]	

Table S8. Categories of automated, unbiased reaction path-searching methods according to the approach of finding elementary reaction steps.

**Categories**	**Method**	**Ref.**
Stationary points searching through predefined rules	Artificial Force Induced Reaction (AFIR) Method, ZStruct method, Stochastic surface walking (SSW) method, autodE, etc.	61–67
Identification of elementary reaction steps by MD simulations	Chemical dynamics simulation (TSSCDS) method, the *ab initio* nanoreactor (ANNR) method, etc.	68–71
Combined method	Combined molecular dynamics and coordinate (MD/CD)	58
......	......	......

Table S9. Selected examples of autonomous discovery of new material and new reactions and potential challenges in the construction of autonomous platforms.[a]

Autonomous discovery	Material and catalyst discovery	72–74
	Automatic reaction process optimization	75–77
	Synthetic route planning and automatic synthesis of drugs	78,79
Challenges and possible solutions	1) Reaction process optimization and *in-situ* monitoring of reactions;<br>2) Automated synthesis of target molecules involving multiple synthetic steps, each with multiple experimental operational scenarios such as allocation of different material forms (solid, liquid) and various post-processing methods (filtration, extraction, column chromatography, etc.);<br>3) Possible solution with deep learning: integration of brain-inspired deep learning algorithms for different functional systems and the realization of closed-loop automated synthesis research. Illustrative application scenarios include motion planning of robots, target recognition and detection in laboratories, automated extraction of chemical domain knowledge (both natural and processed), and holistic solutions to drive the autonomous operation of the entire system.	

[a]For a comprehensive review in this field, please see the reference.[80]

Table S10. Selected examples of deep learning-assisted virtual screening for drug and material discovery.

Type	Examples	Ref.
Drug discovery	Deep Docking: Deep learning-based tool developed to accelerate docking-based virtual screening.	81
Material discovery	Virtual screening of two-dimensional (2D) materials and to accelerate the discovery of new candidates with targeted physical and chemical properties.	82
	Virtual screening of inorganic materials synthesis parameters.	83
	Prescreening of hosts and emitters in deep-blue fluorescent OLEDs.	84

References:

1. Behler, J. & Parrinello, M. Generalized Neural-Network Representation of High-Dimensional Potential-Energy Surfaces. *Phys. Rev. Lett.* **98**, 146401 (2007).
2. Behler, J. Four Generations of High-Dimensional Neural Network Potentials. *Chem. Rev.* **121**, 10037–10072 (2021).
3. Zhang, L., Han, J., Wang, H., Car, R. & Weinan, E. Deep Potential Molecular Dynamics: A Scalable Model with the Accuracy of Quantum Mechanics. *Phys. Rev. Lett.* **120**, 143001 (2018).
4. Wang, H., Zhang, L., Han, J. & E, W. DeePMD-kit: A deep learning package for many-body potential energy representation and molecular dynamics. *Comput. Phys. Commun.* **228**, 178–184 (2018).
5. Schütt, K. T., Sauceda, H. E., Kindermans, P.-J., Tkatchenko, A. & Müller, K.-R. SchNet – A deep learning architecture for molecules and materials. *J. Chem. Phys.* **148**, 241722 (2018).
6. Schütt, K. T. *et al.* SchNetPack: A Deep Learning Toolbox for Atomistic Systems. *J. Chem. Theory Comput.* **15**, 448–455 (2019).
7. Unke, O. T. & Meuwly, M. PhysNet: A Neural Network for Predicting Energies, Forces, Dipole Moments, and Partial Charges. *J. Chem. Theory Comput.* **15**, 3678–3693 (2019).
8. Zubatyuk, R., Smith, J. S., Leszczynski, J. & Isayev, O. Accurate and transferable multitask prediction of chemical properties with an atoms-in-molecules neural network. *Sci. Adv.* **5**, eaav6490 (2019).
9. Yao, K., Herr, J. E. & Parkhill, J. The many-body expansion combined with neural networks. *J. Chem. Phys.* **146**, 014106 (2017).
10. Wang, H. & Yang, W. Force Field for Water Based on Neural Network. *J. Phys. Chem. Lett.* **9**, 3232–3240 (2018).
11. Wang, H. & Yang, W. Toward Building Protein Force Fields by Residue-Based Systematic Molecular Fragmentation and Neural Network. *J. Chem. Theory Comput.* **15**, 1409–1417 (2019).
12. Chen, W.-K., Fang, W.-H. & Cui, G. Integrating Machine Learning with the Multilayer Energy-Based Fragment Method for Excited States of Large Systems. *J. Phys. Chem. Lett.* **10**, 7836–7841 (2019).
13. Wang, Z., Han, Y., Li, J. & He, X. Combining the Fragmentation Approach and Neural Network Potential Energy Surfaces of Fragments for Accurate Calculation of Protein Energy. *J. Phys. Chem. B* **124**, 3027–3035 (2020).
14. Liu, J., Lan, J. & He, X. Toward High-level Machine Learning Potential for Water Based on Quantum Fragmentation and Neural Networks. *J. Phys. Chem. A* **126**, 3926–3936 (2022).
15. Cheng, Z., Zhao, D., Ma, J., Li, W. & Li, S. An On-the-Fly Approach to Construct Generalized Energy-Based Fragmentation Machine Learning Force Fields of Complex Systems. *J. Phys. Chem. A* **124**, 5007–5014 (2020).
16. Cheng, Z. *et al.* Building quantum mechanics quality force fields of proteins with the generalized energy-based fragmentation approach and machine learning. *Phys. Chem. Chem. Phys.* **24**, 1326–1337 (2022).
17. Liao, K., Dong, S., Cheng, Z., Li, W. & Li, S. Combined fragment-based machine learning force field with classical force field and its application in the NMR calculations of macromolecules in solutions. *Phys. Chem. Chem. Phys.* **24**, 18559–18567 (2022).

18. Han, R., Rodríguez-Mayorga, M. & Luber, S. A Machine Learning Approach for MP2 Correlation Energies and Its Application to Organic Compounds. *J. Chem. Theory Comput.* **17**, 777–790 (2021).
19. Schütt, K. T., Gastegger, M., Tkatchenko, A., Müller, K.-R. & Maurer, R. J. Unifying machine learning and quantum chemistry with a deep neural network for molecular wavefunctions. *Nat. Commun.* **10**, 5024 (2019).
20. Yang, W. & Lee, T. S. A density-matrix divide-and-conquer approach for electronic structure calculations of large molecules. *J. Chem. Phys.* **103**, 5674–5678 (1995).
21. Kobayashi, M., Imamura, Y. & Nakai, H. Alternative linear-scaling methodology for the second-order Møller-Plesset perturbation calculation based on the divide-and-conquer method. *J. Chem. Phys.* **127**, 074103 (2007).
22. Kobayashi, M. & Nakai, H. Divide-and-conquer-based linear-scaling approach for traditional and renormalized coupled cluster methods with single, double, and noniterative triple excitations. *J. Chem. Phys.* **131**, 114108 (2009).
23. Makowski, M., Korchowiec, J., Gu, F. L. & Aoki, Y. Describing electron correlation effects in the framework of the elongation method—Elongation-MP2: Formalism, implementation and efficiency. *J. Comput. Chem.* **31**, 1733–1740 (2010).
24. Li, W., Li, S. & Jiang, Y. Generalized energy-based fragmentation approach for computing the ground-state energies and properties of large molecules. *J. Phys. Chem. A* **111**, 2193–2199 (2007).
25. Li, W., Dong, H., Ma, J. & Li, S. Structures and Spectroscopic Properties of Large Molecules and Condensed-Phase Systems Predicted by Generalized Energy-Based Fragmentation Approach. *Acc. Chem. Res.* **54**, 169–181 (2021).
26. Collins, M. A. & Bettens, R. P. A. Energy-Based Molecular Fragmentation Methods. *Chem. Rev.* **115**, 5607–5642 (2015).
27. Gadre, S. R., Yeole, S. D. & Sahu, N. Quantum chemical investigations on molecular clusters. *Chem. Rev.* **114**, 12132–12173 (2014).
28. Liu, J. & He, X. Recent advances in quantum fragmentation approaches to complex molecular and condensed-phase systems. *WIREs Comput. Mol. Sci.* **13**, e1650 (2023).
29. Liu, J. & He, X. Fragment-based quantum mechanical approach to biomolecules, molecular clusters, molecular crystals and liquids. *Phys. Chem. Chem. Phys.* **22**, 12341–12367 (2020).
30. Raghavachari, K. & Saha, A. Accurate Composite and Fragment-Based Quantum Chemical Models for Large Molecules. *Chem. Rev.* **115**, 5643–5677 (2015).
31. Herbert, J. M. Fantasy versus reality in fragment-based quantum chemistry. *J. Chem. Phys.* **151**, 170901 (2019).
32. Pulay, P. Localizability of dynamic electron correlation. *Chem. Phys. Lett.* **100**, 151–154 (1983).
33. Hampel, C. & Werner, H. Local treatment of electron correlation in coupled cluster theory. *J. Chem. Phys.* **104**, 6286–6297 (1996).
34. Li, S., Ma, J. & Jiang, Y. Linear scaling local correlation approach for solving the coupled cluster equations of large systems. *J. Comput. Chem.* **23**, 237–244 (2002).
35. Li, S., Shen, J., Li, W. & Jiang, Y. An efficient implementation of the 'cluster-in-molecule' approach for local electron correlation calculations. *J. Chem. Phys.* **125**, 074109 (2006).
36. Wang, Y. *et al.* Cluster-in-Molecule Method Combined with the Domain-Based Local Pair

Natural Orbital Approach for Electron Correlation Calculations of Periodic Systems. *J. Chem. Theory Comput.* **18**, 6510–6521 (2022).
37. Li, W., Piecuch, P., Gour, J. R. & Li, S. Local correlation calculations using standard and renormalized coupled-cluster approaches. *J. Chem. Phys.* **131**, 114109 (2009).
38. Guo, Y. *et al.* Communication: An improved linear scaling perturbative triples correction for the domain based local pair-natural orbital based singles and doubles coupled cluster method [DLPNO-CCSD(T)]. *J. Chem. Phys.* **148**, 011101 (2018).
39. Pinski, P. & Neese, F. Analytical gradient for the domain-based local pair natural orbital second order Møller-Plesset perturbation theory method (DLPNO-MP2). *J. Chem. Phys.* **150**, 164102 (2019).
40. Ma, Q. & Werner, H.-J. Explicitly correlated local coupled-cluster methods using pair natural orbitals. *WIREs Comput. Mol. Sci.* **8**, e1371 (2018).
41. Yang, J., Chan, G. K.-L., Manby, F. R., Schütz, M. & Werner, H.-J. The orbital-specific-virtual local coupled cluster singles and doubles method. *J. Chem. Phys.* **136**, 144105 (2012).
42. Friedrich, J., Hanrath, M. & Dolg, M. Fully automated implementation of the incremental scheme: Application to CCSD energies for hydrocarbons and transition metal compounds. *J. Chem. Phys.* **126**, 154110 (2007).
43. Friedrich, J. Localized orbitals for incremental evaluations of the correlation energy within the domain-specific basis set approach. *J. Chem. Theory Comput.* **6**, 1834–1842 (2010).
44. Rolik, Z., Szegedy, L., Ladjánszki, I., Ladóczki, B. & Kállay, M. An efficient linear-scaling CCSD(T) method based on local natural orbitals. *J. Chem. Phys.* **139**, 94105 (2013).
45. Nagy, P. R., Samu, G. & Kállay, M. Optimization of the Linear-Scaling Local Natural Orbital CCSD(T) Method: Improved Algorithm and Benchmark Applications. *J. Chem. Theory Comput.* **14**, 4193–4215 (2018).
46. Kjaergaard, T., Baudin, P., Bykov, D., Kristensen, K. & Jørgensen, P. The divide-expand-consolidate coupled cluster scheme. *WIREs Comput. Mol. Sci.* **7**, e1319 (2017).
47. Deringer, V. L. *et al.* Gaussian Process Regression for Materials and Molecules. *Chem. Rev.* **121**, 10073–10141 (2021).
48. Mater, A. C. & Coote, M. L. Deep Learning in Chemistry. *J. Chem. Inf. Model.* **59**, 2545–2559 (2019).
49. Musil, F. *et al.* Physics-Inspired Structural Representations for Molecules and Materials. *Chem. Rev.* **121**, 9759–9815 (2021).
50. Runge, E. & Gross, E. K. U. Density-functional theory for time-dependent systems. *Phys. Rev. Lett.* **52**, 997–1000 (1984).
51. Casida, M. E. & Huix-Rotllant, M. Progress in Time-Dependent Density-Functional Theory. *Annu. Rev. Phys. Chem.* **63**, 287–323 (2012).
52. Foresman, J. B., Head-Gordon, M., Pople, J. A. & Frisch, M. J. Toward a systematic molecular orbital theory for excited states. *J. Phys. Chem.* **96**, 135–149 (1992).
53. Roos, B. O., Taylor, P. R. & Sigbahn, P. E. M. A complete active space SCF method (CASSCF) using a density matrix formulated super-CI approach. *Chem. Phys.* **48**, 157–173 (1980).
54. Werner, H. & Reinsch, E. The self-consistent electron pairs method for multiconfiguration reference state functions. *J. Chem. Phys.* **76**, 3144–3156 (1982).
55. Westermayr, J. & Marquetand, P. Machine Learning for Electronically Excited States of

Molecules. *Chem. Rev.* **121**, 9873–9926 (2021).
56. Westermayr, J. *et al.* Machine learning enables long time scale molecular photodynamics simulations. *Chem. Sci.* **10**, 8100 (2019).
57. Valsson, O., Tiwary, P. & Parrinello, M. Enhancing Important Fluctuations: Rare Events and Metadynamics from a Conceptual Viewpoint. *Annu. Rev. Phys. Chem.* **67**, 159–184 (2016).
58. Yang, M. *et al.* Combined Molecular Dynamics and Coordinate Driving Method for Automatic Reaction Pathway Search of Reactions in Solution. *J. Chem. Theory Comput.* **14**, 5787–5796 (2018).
59. Zhang, J. *et al.* Deep reinforcement learning of transition states. *Phys. Chem. Chem. Phys.* **23**, 6888–6895 (2021).
60. Dewyer, A. L., Argüelles, A. J. & Zimmerman, P. M. Methods for exploring reaction space in molecular systems. *WIREs Comput. Mol. Sci.* **8**, e1354 (2018).
61. Maeda, S., Harabuchi, Y., Hayashi, H. & Mita, T. Toward Ab Initio Reaction Discovery Using the Artificial Force Induced Reaction Method. *Annu. Rev. Phys. Chem.* **74**, 287–311 (2023).
62. Maeda, S., Harabuchi, Y., Takagi, M., Taketsugu, T. & Morokuma, K. Artificial Force Induced Reaction (AFIR) Method for Exploring Quantum Chemical Potential Energy Surfaces. *Chem. Rec.* **16**, 2232–2248 (2016).
63. Behn, A., Zimmerman, P. M., Bell, A. T. & Head-Gordon, M. Incorporating Linear Synchronous Transit Interpolation into the Growing String Method: Algorithm and Applications. *J. Chem. Theory Comput.* **7**, 4019–4025 (2011).
64. Zimmerman, P. M. Automated discovery of chemically reasonable elementary reaction steps. *J. Comput. Chem.* **34**, 1385–1392 (2013).
65. Shang, C. & Liu, Z.-P. Stochastic Surface Walking Method for Structure Prediction and Pathway Searching. *J. Chem. Theory Comput.* **9**, 1838–1845 (2013).
66. Kang, P.-L., Shang, C. & Liu, Z.-P. Large-Scale Atomic Simulation via Machine Learning Potentials Constructed by Global Potential Energy Surface Exploration. *Acc. Chem. Res.* **53**, 2119–2129 (2020).
67. Young, T. A., Silcock, J. J., Sterling, A. J. & Duarte, F. autodE: Automated Calculation of Reaction Energy Profiles— Application to Organic and Organometallic Reactions. *Angew. Chemie Int. Ed.* **60**, 4266–4274 (2021).
68. Martínez-Núñez, E. An automated method to find transition states using chemical dynamics simulations. *J. Comput. Chem.* **36**, 222–234 (2015).
69. Martínez-Núñez, E. An automated transition state search using classical trajectories initialized at multiple minima. *Phys. Chem. Chem. Phys.* **17**, 14912–14921 (2015).
70. Wang, L.-P. *et al.* Discovering chemistry with an ab initio nanoreactor. *Nat. Chem.* **6**, 1044–1048 (2014).
71. Cui, Q., Peng, J., Xu, C. & Lan, Z. Automatic Approach to Explore the Multireaction Mechanism for Medium-Sized Bimolecular Reactions via Collision Dynamics Simulations and Transition State Searches. *J. Chem. Theory Comput.* **18**, 910–924 (2022).
72. Zhu, Q. *et al.* An all-round AI-Chemist with a scientific mind. *Natl. Sci. Rev.* **9**, nwac190 (2022).
73. Burger, B. *et al.* A mobile robotic chemist. *Nature* **583**, 237–241 (2020).
74. Xie, M. *et al.* Fast Screening for Copper-Based Bimetallic Electrocatalysts: Efficient Electrocatalytic Reduction of CO2 to C2+ Products on Magnesium-Modified Copper. *Angew.*

Chemie Int. Ed. **61**, e202213423 (2022).
75. Shields, B. J. *et al.* Bayesian reaction optimization as a tool for chemical synthesis. *Nature* **590**, 89–96 (2021).
76. Ahneman, D. T., Estrada, J. G., Lin, S., Dreher, S. D. & Doyle, A. G. Predicting reaction performance in C–N cross-coupling using machine learning. *Science (80-.).* **360**, 186–190 (2018).
77. Zahrt, A. F. *et al.* Prediction of higher-selectivity catalysts by computer-driven workflow and machine learning. *Science* **363**, eaau5631 (2019).
78. Coley, C. W. *et al.* A robotic platform for flow synthesis of organic compounds informed by AI planning. *Science* **365**, eaax1566 (2019).
79. Klucznik, T. *et al.* Efficient Syntheses of Diverse, Medicinally Relevant Targets Planned by Computer and Executed in the Laboratory. *Chem* **4**, 522–532 (2018).
80. Wang, G. *et al.* Machine Learning in Unmanned Systems for Chemical Synthesis. *Molecules* **28**, 2322 (2023).
81. Gentile, F. *et al.* Deep Docking: A Deep Learning Platform for Augmentation of Structure Based Drug Discovery. *ACS Cent. Sci.* **6**, 939–949 (2020).
82. Cihan Sorkun, M. C. *et al.* An artificial intelligence-aided virtual screening recipe for two-dimensional materials discovery. *npj Comput. Mater.* **6**, 106 (2020).
83. Kim, E. *et al.* Virtual screening of inorganic materials synthesis parameters with deep learning. *npj Comput. Mater.* **3**, 53 (2017).
84. Jeong, M. *et al.* Deep learning for development of organic optoelectronic devices: efficient prescreening of hosts and emitters in deep-blue fluorescent OLEDs. *npj Comput. Mater.* **8**, 147 (2022).
